# The microRNA processing subunit DGCR8 is required for a T cell-dependent germinal center response

**DOI:** 10.3389/fimmu.2022.991347

**Published:** 2022-12-16

**Authors:** Patrick Daum, Shannon R. Ottmann, Julia Meinzinger, Sebastian R. Schulz, Joana Côrte-Real, Manuela Hauke, Edith Roth, Wolfgang Schuh, Dirk Mielenz, Hans-Martin Jäck, Katharina Pracht

**Affiliations:** Division of Molecular Immunology, Internal Medicine III, Nikolaus-Fiebiger-Center of Molecular Medicine, Friedrich-Alexander University Erlangen-Nürnberg and University Hospital Erlangen, Erlangen, Germany

**Keywords:** DGCR8, DiGeorge syndrome critical region 8 protein, microRNA, B1 cell, plasma cell (PC), antibody-secreting cell, germinal center response

## Abstract

We have previously shown that the microRNA (miRNA) processor complex consisting of the RNAse Drosha and the DiGeorge Critical Region (DGCR) 8 protein is essential for B cell maturation. To determine whether miRNA processing is required to initiate T cell-mediated antibody responses, we deleted DGCR8 in maturing B2 cells by crossing a mouse with loxP-flanked DGCR8 alleles with a CD23-Cre mouse. As expected, non-immunized mice showed reduced numbers of mature B2 cells and IgG-secreting cells and diminished serum IgG titers. In accordance, germinal centers and antigen-specific IgG-secreting cells were absent in mice immunized with T-dependent antigens. Therefore, DGCR8 is required to mount an efficient T-dependent antibody response. However, DGCR8 deletion in B1 cells was incomplete, resulting in unaltered B1 cell numbers and normal IgM and IgA titers in DGCR8-knock-out mice. Therefore, this mouse model could be used to analyze B1 responses in the absence of functional B2 cells.

## Introduction

MicroRNAs (miRNAs) are small non-coding single-stranded epigenetic regulators initially found to control the larva development of *Caenorhabditis elegans* (1). Higher eukaryotes use miRNAs to regulate gene expression at the post-transcriptional level (2), and altered miRNA expression is associated with many diseases (3, 4).

Canonical miRNA maturation begins in the nucleus by synthesizing long primary transcripts (pri-miRNA) (5). pri-mRNAs are processed to a ~70 nucleotide long pre-miRNA duplex with a lariat structure by the heterotrimeric microprocessor complex consisting of the RNA-binding protein DGCR8 (*DiGeorge syndrome chromosomal/critical region 8*) and the RNAse III Drosha (6, 7). Next, pre-miRNAs are exported into the cytoplasm by exportin-5, where they are further processed by the RNAse DICER1-TRBP complex to a short double-stranded RNA molecule (8). The leading strand of this duplex is integrated into the *RNA-induced silencing complex* (RISC). The mature miRNA then guides the RISC to its target mRNA, which results in its degradation or inhibition of translation.

Studies analyzing the effect of targeted deletion of Dicer (9), DGCR8 (10), as well as members of the Argonaut-protein family (11) showed that these components of the miRNA processing machinery are essential in a variety of tissues. For example, B cell-specific ablation of Dicer or DGCR8 in early B cell precursors resulted in a developmental block at the pro-B cell stage (12, 13). Furthermore, Dicer deficiency in germinal center (GC) B cells revealed an impairment of GC formation and T-dependent (TD) antibody responses (14). In contrast, the conditional CD19-Cre-mediated deletion of Dicer resulted in an altered B cell receptor (BCR) repertoire and high serum titers of auto-antibodies (15).

To investigate the role of DGCR8 and the canonical miRNA processing pathway in establishing the mature B2 cell population consisting of follicular (FO) and marginal zone (MZ) B cells and their antigen-dependent activation, DGCR8 deletion was induced during the maturation process of splenic transitional B2 cells by crossing a transgenic CD23-Cre mouse (16) to a mouse strain with loxP-flanked DGCR8 alleles (12). We demonstrate that CD23-Cre-mediated DGCR8 deletion in mice impaired the establishment of FO B2 cells and led to a reduction in IgG serum titers and the number of antigen-specific IgG-secreting cells. Furthermore, analysis of the cell viability of *in vitro* generated plasmablasts showed that ablation of mature miRNAs compromised the survival of LPS-activated mature B cells, which could, at least in part, mechanistically explain impaired T-dependent antigen-specific humoral immune response with reduced GC formation.

## Results

### B cell-specific DGCR8-deficiency reduces serum IgG titers and impairs the establishment of mature B cells as well as IgG-secreting cells *in vivo*

To investigate the role of DGCR8 in maturing B2 cells, mice with loxP-flanked (floxed) DGCR8 alleles (12) were crossed to CD23-Cre transgenic mice (16). In this mouse line, the Cre activity is coupled to the expression of the Fc epsilon receptor II (a) promoter, also known as CD23(a). Expression of CD23 is induced in secondary lymphatic organs at transitional stage 2 of immature B cells (T2 and T3 B cells) and in FO B cells (17). T2 B cells are the common precursor of MZ and FO B cells.

**Figure 1:**
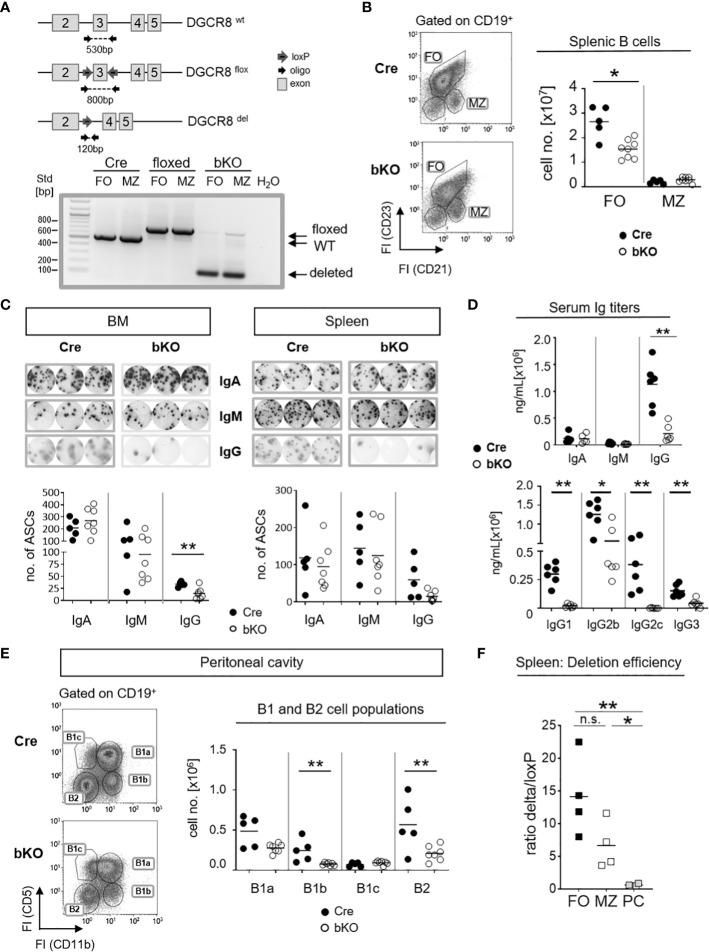
B cell-specific DGCR8 deficiency impairs the establishment of mature B cells and IgG-secreting cells *in vivo*. **(A)** Construction of a mouse carrying a DGCR8 allele with a floxed exon 3 (upper panel). The locus was analyzed by PCR of flow cytometry-sorted MZ B cells (B220^+^CD19^+^CD93^-^CD23^low^CD21^+^) and FO B cells (B220^+^CD19^+^CD93^-^CD23^+^CD21^low^) from DGCR8-bKO, Cre-only and floxed-only control mice, respectively. PCR product/amplicon sizes are ~530 bp for the wildtype, ~800 bp for the floxed and ~120 bp for the Cre-deleted DGCR8 allele. **(B)** Flow cytometry analysis of splenic B cells from DGCR8-bKO (bKO) and Cre control mice. Stained samples were pre-gated on CD19-positive cells. Cell numbers of MZ- and FO B cells in the total spleen cell populations were quantified by flow cytometry using flow count beads. Bars indicate the median of n=5 (Cre) or n=8 (bKO) mice. **(C)** ELISpot assay to quantify IgA, IgM or IgG-secreting cells in the bone marrow (BM; left panel) and the spleen (right panel) from non-immunized DGCR8-bKO and Cre control animals. Depicted wells show the results from a total of 3,33x10^4^ seeded cells. **(D)** Serum samples from non-immunized DGCR8-bKO and Cre control mice of 9-18 weeks in age were analyzed by ELISA for total IgA, IgM, IgG and IgG-subclasses. n=6 mice of each genotype. **(E)** Flow cytometry analysis of samples from peritoneal lavages of DGCR8-bKO and Cre control mice to quantify B1a (CD19^+^CD5^+^CD11b^+^), B1b (CD19^+^CD5^-^CD11b^+^), B1c (CD19^+^CD5^+^CD11b^-^) and B2 (CD19^+^CD5^-^CD11b^-^) cell populations. Cell numbers were calculated per total lavage. **(F)** Cre-mediated DGCR8-deletion efficiency was analyzed by quantifying PCR amplicon abundances of the loxP-flanked and the deleted DGCR8-allele from sorted FO, MZ and CD138^+^TACI^+^- populations of DGCR8-bKO mice (**Figure 1A** and **Supplemental Figure 1A**). Displayed is the ratio between the deleted allele and the loxP-flanked allele in the same sample. Amplicon abundances were quantified by ImageJ. Bars indicate the median of **(B-E)** n=5 (Cre) or n=7 (bKO) mice and **(F)** n=4 mice. Each dot represents one mouse. Mann-Whitney test was used for statistical analysis. **p<0.01; * p<0.05, n.s., not significant.

To test the DGCR8 deletion efficiency in mature B2 cells, genomic DNA from splenic MZ (CD19^+^CD21^+^CD23^low^)- and FO (CD19^+^CD21^low^CD23^+^) B cells of CD23-Cre^+/-^ DGCR8^fl/fl^ mice (DGCR8-bKO), CD23-Cre^-/-^ DGCR8^fl/fl^ (floxed) and CD23-Cre^+/-^ DGCR8^wt/wt^ (Cre) littermates was PCR amplified with primers flanking the floxed exon 3 of the DGCR8 gene locus (**Figure 1A**). As expected, CD23-Cre-mediated DGCR8 deletion was efficient in FO and MZ B cells (**Figures 1A, F**). Therefore, DGCR8-bKO mice were used to analyze the role of mature miRNAs in establishing the splenic B2 cell populations and their T-dependent antigen-driven activation.

To analyze if DGCR8 loss affects the development of mature splenic B cell populations *in vivo*, we investigated non-immunized DGCR8-bKO and Cre control mice. Flow cytometric analysis of DGCR8-bKO mice revealed a significant decline (~1.7 fold) of splenic FO B cell numbers. In contrast, the MZ B cell population was not significantly altered (**Figure 1B**).

Since FO B cells comprise the majority of the mature B2 cell population and are mainly contributing to the generation of T-dependent antigen-specific humoral immunity (18), we speculated that the decreased number of FO B cells (**Figure 1B**) impacts the number of IgH class-switched antibody-secreting cells (ASC). As expected, ELISpot analyses revealed an apparent decrease in IgG-positive ASCs in bone marrow (BM, significant) and spleen (not significant) of non-immunized DGCR8-bKO mice compared to Cre control mice (**Figure 1C**). However, the numbers of IgM- and IgA-positive ASCs were not altered in both organs of DGCR8-bKO mice (**Figure 1C**).

The effect of DGCR8 ablation on the development of IgG-secreting cells (**Figure 1C**) was verified by ELISA in sera of DGCR8-bKO mice, i.e., a severe reduction in serum IgG (~4.9x fold decrease) was observed in DGCR8-bKO mice. In contrast, total serum IgM and IgA concentrations were unaltered (**Figure 1D**). Interestingly, while concentrations of IgG2b and IgG3 were reduced but could still be detected in the serum of DGCR8-bKO mice, IgG1 and IgG2c were completely undetectable.

IgG2b and IgG3 subclasses are secreted by *in vitro* stimulated B1 cells (19, 20). In addition, B1a cells develop from CD23-negative precursors, are self-renewing and provide most of the natural IgM and half of the serum IgA (21). In support, the floxed DGCR8 allele could still be detected by PCR from DNA isolated from peritoneal B1a cells of DGCR8-bKO mice (**Supplemental Figure 1B**). Therefore, it is tempting to speculate that most of the serum IgA and IgM and the detectable IgG subclasses in DGCR8-bKO mice are provided by plasma cells originating from CD23-negative B1a cells (22). To address this hypothesis, we examined B1 cell populations in the preferred location, i.e., the peritoneal cavity (23). As expected, flow cytometry analyses revealed that bone marrow-derived B1b and B2 cells were significantly reduced in the peritoneal cavity of DGCR8-bKO animals. In contrast, the numbers of fetal liver-derived B1a and B1c cells were not significantly altered (**Figure 1E**). In addition, B1a cells can also reside in the spleen and the bone marrow (24). As expected, B1 cell numbers were unaltered in the spleen of DGCR8-bKO mice (**Supplemental Figure 2A**), which at least partially explains the detection of IgM- and IgA-secreting cells in these tissues (**Figure 1C**). Hence, unaltered B1 cell numbers in the peritoneal cavity and spleen of DGCR8-bKO mice can provide serum IgM, IgA, and presumably, IgG2b and IgG3 detected in non-immunized DGCR8-bKO mice (**Figure 1D**). Furthermore, the Cre-mediated deletion of the loxP-flanked DGCR8 exon 3 resulted in an efficient deletion in FO B cells, but a significant increase in DGCR8 non-deleted cells could be detected among CD138^+^TACI^+^ ASCs (**Figure 1F** and **Supplemental Figure 1A**). This data supports the hypothesis that the ASC pool in DGCR8-bKO mice mainly originates from B1a cells, further supporting the pivotal role of DGCR8-dependent miRNAs in the formation of B2-derived ASCs.

### DGCR8 is essential for germinal center formation

**Figure 2:**
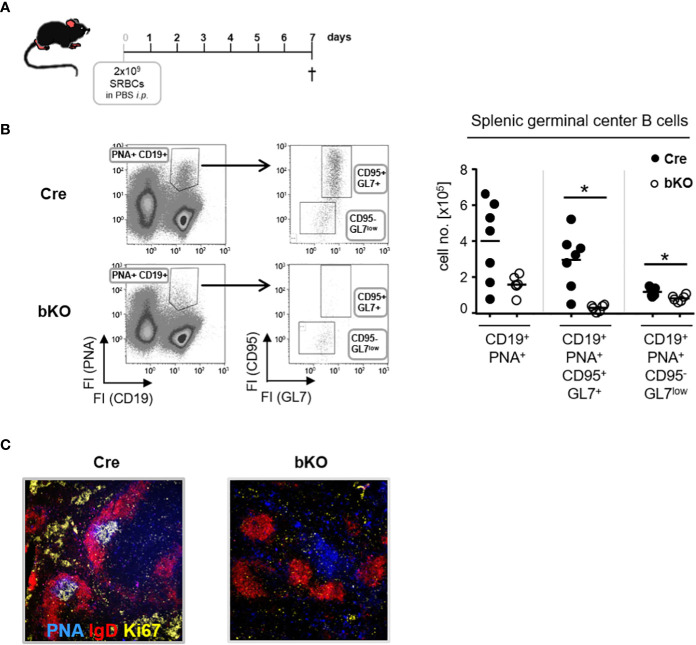
DGCR8 is essential for germinal center formation. **(A)** DGCR8-bKO mice and Cre control animals were immunized with sheep red blood cells (SRBC) and analyzed one week later. **(B)** Flow cytometric analysis of splenic cells from DGCR8-bKO mice and Cre control animals treated in **(A)**. GC B cells were defined as CD19^+^PNA^+^ and further subdivided into CD95^+^GL7^+^ and CD95^-^GL7^low^ cells. Cell numbers were calculated for the whole spleen. n=6-7 mice per genotype. **(C)** Immunofluorescence microscopy of splenic cryosections from mice treated in **(A)**. Sections were stained for IgD (red), PNA (blue), and Ki67 (yellow) to visualize the FO mantle zone (IgD^+^), as well as the light- and dark zone of the GC (PNA^+^ or Ki67^+^). Each dot represents a mouse and bars indicate the mean of all mice with the same genotype. The Mann-Whitney test was used for statistical analysis. *p<0.05.

To determine whether DGCR8 ablation in T2-originated B2 cells affects the T-dependent (TD) germinal center (GC) formation and, therefore, IgG serum titers and numbers of IgG-secreting cells, we induced a robust TD-immune response by injecting mice with sheep red blood cells (SRBC). After 7 days, mice were sacrificed, and GC formation was analyzed (**Figure 2A**). Flow cytometric analysis of splenic cells from DGCR8-bKO mice showed an apparent but non-significant reduction in the numbers of CD19^+^PNA^+^ GC B cells compared to Cre control animals (**Figure 2B**). GC B cells were further analyzed for expression of GL-7 and CD95, two additional markers separating cycling GC B cells and more mature GC B cells already primed to differentiate into plasmablasts (25). Dividing GC B cells (CD95^+^GL-7^+^) were significantly reduced in DGCR8-bKO mice (11-fold), whereas the cell population destined for plasmablast differentiation (CD95^-^GL-7^low^) was slightly but significantly diminished (1.4-fold). Histological analysis from the same spleens confirmed the inability of DGCR8-bKO mice to form morphologically intact GCs (**Figure 2C**). Contrary to Cre control animals, DGCR8-bKO mice were not able to develop the GC characteristic dark- (Ki67^+^) or light zone (PNA^+^) within the mantle zone (IgD^+^) of the B cell follicle.

Based on these findings, we conclude that CD23-Cre-mediated ablation of DGCR8 in transitional B cells severely affects the establishment of GCs, which explains the reduced serum IgG titers and diminished numbers of IgG-secreting cells in DGCR8-bKO mice (**Figure 1**).

### DGCR8-bKO mice fail to mount a TD antigen-specific IgG response

We could demonstrate that DGCR8-bKO mice resemble a phenotype characteristic for hypogammaglobulinemia, with reduced basal levels of serum IgG, diminished numbers of IgG-secreting cells and disturbed formation of GC B cells and structures (**Figures 1C, D**, **2**).

**Figure 3:**
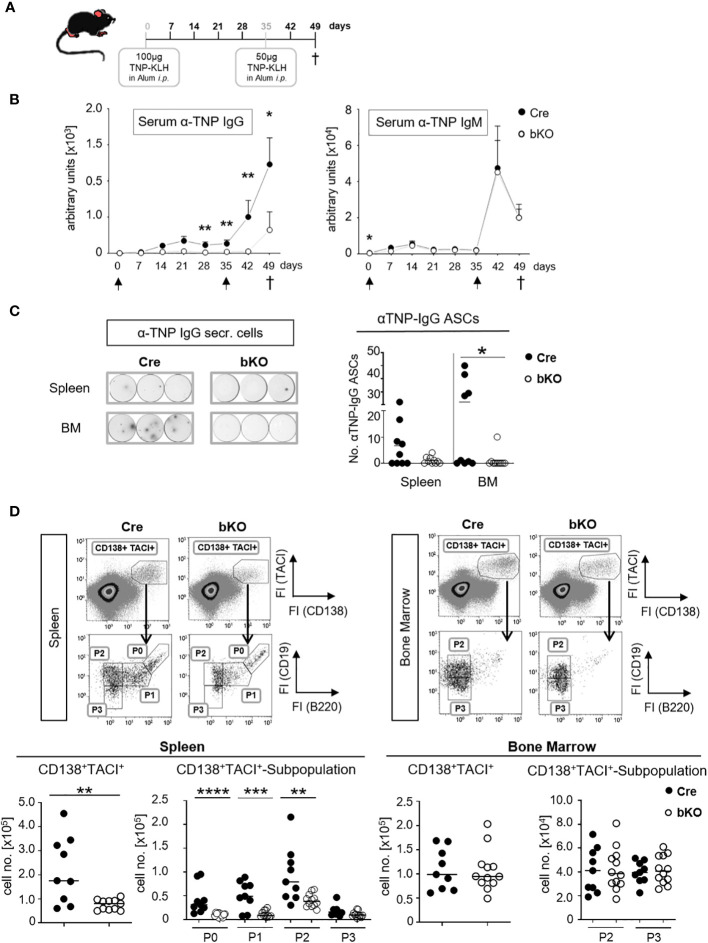
CD23-Cre-mediated DGCR8-deficient mice fail to mount a TD antigen-specific IgG response. **(A)** DGCR8-bKO and Cre control mice (9-18 weeks in age) were injected intraperitoneally with TNP-KLH in alum, boostered on day 35 and sacrificed 49 days after primary immunization. **(B)** Blood was collected weekly, and sera were analyzed for TNP-specific IgG and IgM by ELISA. Arrows indicate immunization time points. Each dot represents the mean (+SEM) of n=9 (Cre) or n=12 (bKO) mice. **(C)** Mice were sacrificed on day 49 p.i. (post-immunization), spleen and bone marrow (BM) cell suspensions were analyzed for TNP-specific IgG-secreting cells by ELISpot assay. The examples show the results obtained from 6,67x10^5^ cells incubated per well. Cell numbers are calculated per 2x10^6^ seeded cells. **(D)** Flow cytometric analysis of splenic and bone marrow cells. Antibody-secreting cells were defined as CD138^+^ TACI^+^ cells and further divided by CD19 and B220 surface abundances in P0-activated B cells, P1-plasmablasts, P2-early plasma cells and P3-mature plasma cells (26). Cell numbers were calculated for one tibia and one femur or the entire spleen. **(C, D)** Bars indicate the median of n=9 (Cre) or n=12 (bKO) mice from N=3 experiments. Each dot represents an individual mouse. Maximal one significant outlier per genotype and analysis was removed from the analysis (Grubb’s test). The Mann-Whitney test was used for statistical analysis if the Shapiro-Wilk test showed no Gaussian distribution; otherwise, the t-test was used. *p<0.05; ** p<0.01; *** p<0.005; **** p<0.001.

To determine whether DGCR8-bKO mice generate an antibody response to a TD antigen, mice were immunized with TNP-KLH in alum, boosted with TNP-KLH in alum and finally analyzed on day 49 after primary immunization (**Figure 3A**). Strikingly, DGCR8-bKO mice failed to produce TNP-specific IgG in response to the first immunization, and they responded poorly, if at all, to a booster immunization with the same antigen (**Figure 3B**). In contrast, the production of TNP-specific IgM was not affected in DGCR8-bKO mice and, as discussed before, was likely mounted by B1 cells.

ELISpot analysis showed a severe reduction of TNP-specific IgG-secreting cells in the bone marrow of DGCR8-bKO mice (**Figure 3C**), supporting the serum titer results in **Figure 3B**. Except for one individual mouse, no TNP-specific IgG-secreting cells were detectable in the bone marrow (BM) of DGCR8-bKO mice. A similar trend was observed in the spleens of the same mice; even so, alterations were insignificant. These results are supported by flow cytometry analysis, where we detected significantly reduced numbers of CD138^+^TACI^+^ ASCs in the spleen of DGCR8-bKO mice (**Figure 3D**, left). Among the CD138^+^TACI^+^ cells, especially the numbers of CD19^+^B220^+^ activated B cells (P0) and plasmablasts (P1) and CD19^+^B220^-^ early plasma cells (P2) were significantly reduced, while the numbers of CD19^-^B220^-^ mature plasma cells (P3) were unaltered (26). However, the numbers of CD138^+^TACI^+^ plasma cells (P2 and P3) in the BM of immunized DGCR8-bKO mice did not differ from those of Cre control animals (**Figure 3D**, right), which could be explained by the presence of a functional B1a compartment in DGCR8-bKO mice.

In summary, B2 cell-specific abrogation of DGCR8 by CD23-Cre is associated with a lack of antigen-specific serum IgG (**Figures 1C, D**) and the inability of the mice to generate or maintain IgG-secreting plasma cells upon TD immunization (**Figures 3B, C**). Therefore, we suggest that T cell-dependent GC reactions are disrupted in mice that lack expression of the miRNA processor component DGCR8 in mature B2 cells.

### DGCR8 deficiency affects the viability of follicular and marginal zone B cells *in vitro*

**Figure 4:**
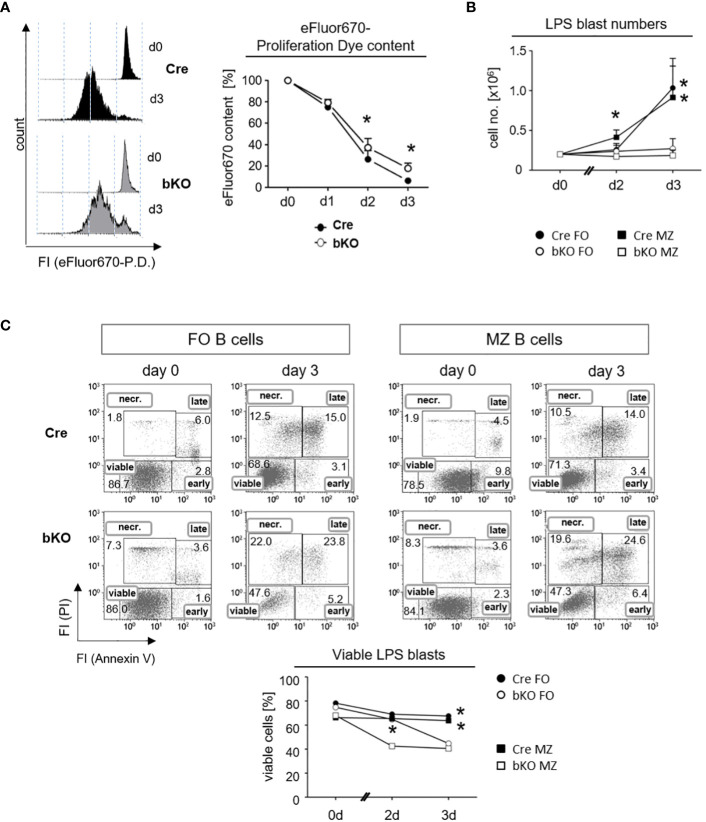
DGCR8-deficiency affects the proliferation and viability of follicular and marginal zone B cells *in vitro.* **(A)** Naive splenic B cells were isolated from Cre control and DGCR8-bKO mice by magnetic cell sorting (EasySep^©^) and analyzed for lipopolysaccharide (LPS)-induced proliferation *in vitro*. Cells were stained with the proliferation dye (P. D.) eFluor670 right after isolation, and the content of the dye was measured for three days using flow cytometry. The representative figure shows eFluor670 content (day 0 and day 3). The graph depicts changes in eFluor670 fluorescence intensity of Cre and DGCR8-bKO cells normalized to the basic value determined on day 0. Points represent the mean from n=4 mice per genotype. **(B)** Fluorescence-based flow cytometry-sorted splenic MZ B cells (CD19^+^CD23^low^CD21^+^) and FO B cells (CD19^+^CD23^+^CD21^low^) from Cre control and DGCR8-bKO mice were stimulated *in vitro* with LPS. Cell numbers were determined using flow count beads. Points represent the mean from n=3 mice per genotype. **(C)** Cell viability in the samples described in **(B)** was analyzed at different time points by flow cytometry with propidium iodide (PI) and AnnexinV. AnnexinV- and PI-negative cells were defined as viable. Mann-Whitney test was used for statistical analysis. * p<0.05; **p<0.01.

A previous study from our lab revealed that the conditional deletion of DGCR8 in early B cell precursors blocked the central maturation of pro-B cells into mature B cells due to the impaired viability of pro-B cells (12). We, therefore, hypothesized that the inability of B2 cells to form a GC reaction in DGCR8-bKO mice (**Figure 2**) could also be explained by a diminished survival potential of DGCR8-negative B cells before the onset of a GC reaction. To evaluate this idea, we labeled freshly isolated splenic B cells from Cre control and DGCR8-bKO mice with a fluorescent proliferation dye and measured the decrease in the fluorescence intensities to determine the number of cell divisions (**Figure 4A**). Upon LPS activation, fluorescence-labeled DGCR8-deficient B cells showed a 2.9-fold higher fluorescence content of the proliferation dye than the control cells three days after activation, indicating retardation in cell proliferation.

To specifically address the effect of DGCR8-deficiency on FO and MZ B cells, we isolated these splenic B cell populations by fluorescence-activated cell sorting (FACS) and analyzed them *in vitro* for their viability by an AnnexinV/propidium iodide (PI)-assay under the same stimulatory conditions as described in **Figure 4A**. First, we observed that LPS activation resulted in no expansion of cell numbers in cultures of DGCR8-deficient FO B cells and MZ B cells. In contrast, B cells from Cre control animals expanded as expected in response to LPS (**Figure 4B**). Furthermore, the viability of the isolated DGCR8-deficient FO and MZ B cells decreased over time, showing 20% fewer AnnexinV/PI-negative viable cells compared to Cre controls after three days in culture (**Figure 4C**).

These results imply that DGCR8 ablation significantly impaired B cell proliferation upon stimulation but had an even more significant impact on cell viability. This explains the dramatic restriction in activated B cell expansion *in vitro*. Moreover, it is still unclear if a survival defect in DGCR8-bKO mice occurs early in B cell activation or during the establishment of GCs *in vivo*. Nevertheless, the diminished survival potential of B cells upon antigen activation could explain the defect in GC formation (**Figure 2**) and the inability to mount a highly specific IgG-response (**Figure 3**) in immunized DGCR8-bKO mice.

## Discussion

In this study, we demonstrate in mice with a B cell-specific conditional DGCR8 deficiency (DGCR8-bKO) that the ablation of microRNAs in maturing B2 cells drastically affects the humoral immune response before the onset of GCs.

The decreased viability of DGCR8-deficient FO B cells *in vitro* was in line with the observation of a diminished FO B cell population in the spleens of DGCR8-bKO mice. This result is in accordance with the reduced survival of Mb1-Cre-mediated DGCR8-deficient pro-B cells (12). Although isolated splenic DGCR8-deficient MZ B cells exhibit defective survival upon stimulation *in vitro*, this population was not altered in cell numbers *in vivo*. Therefore, we assume that residual DGCR8 mRNA and mature miRNAs in MZ B cells might temporally bypass the deletion of the DGCR8 allele and mask a potential phenotype *in vivo* (**Supplemental Figure 1**). Furthermore, analysis for apoptotic cells within the freshly isolated B cell subsets showed no significant differences between the mice of both genotypes, with just a small increase in apoptotic cells within the GC B cell subset of DGCR8-bKO mesenteric lymph nodes (**Supplemental Figure 2C**). However, reliable quantification of apoptotic cells on freshly isolated tissues is challenging, as apoptotic cells are quickly cleared by, e.g., macrophages in the living organism.

Furthermore, mRNA abundance of the pro-apoptotic factor Bim was negligible in all analyzed freshly isolated B cell samples, showing unaltered Bim expression in splenic FO B cells of DGCR8-bKO mice and even reduced expression in MZ B cells (**Supplemental Figure 1C**). These results support the hypothesis of fast clearance of apoptotic cells *in vivo*. In addition, intercellular interactions, like the secretion of B cell-activating factor (BAFF) by lymphoid cells, could temporarily suppress apoptotic processes in DGCR8-deficient B cells *in vivo* (27), as BAFF receptor (BAFF-R) abundance was unaltered on B cell populations from spleen and mesenteric lymph nodes of DGCR8-bKO mice (**Supplemental Figure 2B**). As Aicda-Cre mediated deletion of Dicer results in a loss of mature miRNAs from the canonical pathway and a complete abolishment of the GC response (14), miRNAs seem to be essential for GC B cells. Whether CD23-Cre-mediated deletion of DGCR8 and earlier abolishment of canonical miRNA maturation in mature B cells already dampens primary B cell activation or affects GC B cells comparable to the phenotype in Aicda-Cre Dicer knock-outs is still unclear.

Nevertheless, analysis of B cell populations in non-immunized DGCR8-bKO mice implies that DGCR8 ablation in maturing B cells has a functional effect on the generation of ASCs. In particular, the class switching to IgG-secreting cells and, thereby, the production of IgG is affected in DGCR8-bKO mice. Flow cytometry and fluorescence microscopy revealed that B cells could not form morphologically intact GCs without mature miRNAs. Surprisingly, the formation of IgA- or IgM-secreting cells was not significantly compromised in DGCR8-bKO mice. In addition, IgG2b and IgG3 secretion were reduced, while other IgG-subclasses were absent in the serum of DGCR8-bKO mice. This particular phenotype is of most significant interest, as B1 cells can switch to Ig-secreting cells of those IgH subclasses that were just reduced in DGCR8-bKO mice (19–21). Furthermore, numbers of CD23-negative self-renewing B1a cells in the peritoneal cavity and spleen, originating from fetal liver cells (22), were as expected unaffected in DGCR8-bKO mice (**Figure 1E** and **Supplemental Figure 2A**). The presence of a functional B1 compartment was supported by our findings that DGCR8 deletion was incomplete in isolated B1a cells from the peritoneal cavity (**Supplemental Figure 1B**). Therefore, the humoral immune response observed in DGCR8-bKO mice is likely mounted by B1 cells.

We observed that IgA and IgM are the predominant IgH isotypes in non-immunized mice kept in our local animal facility. At the same time, IgG-secreting cells only represent a minor fraction of the total plasma cell population in the BM (**Figure 1C**). Accordingly, due to their low frequencies, the loss of IgG-secreting cells in the BM of DGCR8-bKO mice very likely does not affect the total number of plasma cells, which explains the unaltered numbers of BM CD138^+^TACI^+^ plasma cells in DGCR8-bKO mice compared to Cre controls (**Figure 1D**). Furthermore, only small numbers of plasma cells are usually found in the bone marrow (28); consequently, survival niches could be populated by non-IgG-secreting plasma cells that originated from B1 cells in DGCR8-bKO mice. Our PCR data support this hypothesis, showing increased frequencies of DGCR8 non-deleted ASCs compared to FO or MZ B cells in DGCR8-bKO mice (**Figure 1F** and **Supplemental Figure 1A**). In addition, numbers of mature CD138^+^TACI^+^ P3 plasma cells were unaltered in the spleen of DGCR8-bKO mice, while the numbers of activated B cells and plasmablasts were significantly reduced (**Figure 1D**). This data supports that DGCR8 ablation in mature B2 cells results in a severely reduced germinal center output, while ASCs from B1 origin can populate survival niches.

Taking into consideration that DGCR8-bKO mice did not exhibit an adequate GC formation upon activation with TD-antigen, it is surprising that these mice can mount an antigen-specific IgM memory-like response (against TNP), as documented in **Figure 3B**. However, several studies reported that memory B cells could originate from a GC-independent pathway early during an immune response (29–31). Furthermore, IgM+ memory B cells are likely generated in a GC-independent manner (32, 33) and can also originate from B1 cells (34). In addition, as hypothesized before, most of the serum IgM and IgA detected in DGCR8-bKO mice could originate from B1 cells as these cells can undergo a T-independent IgH-chain class switch comparable to B2 cells (35, 36). Therefore, these findings explain the existence of an antigen-specific IgM memory response and the formation of the antigen-specific IgM titers, which we observed in DGCR8-bKO mice.

Our study demonstrated that the deletion of DGCR8 in maturing B2 precursors almost completely abolished a TD GC reaction. As the CD23-Cre-mediated deletion of DGCR8 is incomplete in B1a cells, this B cell compartment might be responsible for most of the antibody responses observed in DGCR8-bKO mice. Thus, the CD23-Cre/DGCR8 KO mouse could be a unique and excellent model to study B1a responses and tumorigenic events without a functional FO and MZ B cell compartment.

## Material and methods

### Mice

All mice were maintained under pathogen-free conditions in the Nikolaus-Fiebiger Center animal facility of the University of Erlangen-Nürnberg, Erlangen, Germany. All animal experiments were performed according to institutional and national guidelines. Transgenic CD23-Cre mice (16) were crossed with loxP-flanked DGCR8-mice (12). The mice have a C57Bl/6 background. We used age and sex-matched mice of both sexes for all analyses. Mice were 9-18 weeks old for steady state analyses.

### Immunization of mice

Mice were immunized with 100 µg (100 µl in PBS) TNP-KLH (load 18, LGC Biosearch Technologies) in 100 µl alum (Imject Alum Adjuvants, ThermoFisher) intraperitoneally. On day 35, mice were boosted with 50 µg (50 µl) TNP-KLH in 50 µl alum intraperitoneally. Alternatively, mice were immunized with 2x10^9^ sheep red blood cells (SRBC, Fiebig Nährstofftechnik) in 300 µl PBS intraperitoneally. Mice were 9-18 weeks old at the time of the first immunization.

### Isolation of murine cells

BM cells were obtained by opening (removing joints) and flushing the hollow bones (femur and tibia), spleens were minced and passed through a cell strainer (70 μm, Falcon, Cat# 352350), and blood was obtained by cardiac puncture. Cells were washed with PBS/2% FCS or R10^+^ medium (Gibco: 500 mL RPMI1640 Cat# 31870-25; 2mM L-glutamine Cat# 25030-24; 1 mM sodium pyruvate Cat# 11360-039; 100 U/mL penicillin/streptomycin Cat# 15140-122; 0.05μM β-mercaptoethanol Cat# 31350-010; 50 mL heat-inactivated fetal bovine serum (FCS) Cat# 10270-106) and centrifuged at 470g for 7 min at 4°C. Red blood cells (RBC) in the cell pellets from all analyzed organs (except mesenteric lymph nodes) were lysed in RBC lysis buffer (either 0.15 M NH4Cl/0.02 M Hepes solution or RBC lysis buffer from Biolegend, Cat# 420301) for 6 min at RT. The lysis was stopped by adding PBS/2% FCS or R10 medium. Cells were filtered to remove clotted substances (30μm, Sysmex, Cat# 04-0042-2316), centrifuged (470g, 7 min, 4°C), and suspended in the desired volume PBS/2% FCS or R10 medium. Cell concentrations were determined in a Neubauer counting chamber or a Nucleocounter NC3000 (ChemoMetec, Allerod, Denmark).

### Flow cytometry analysis

1-2x10^6^ isolated cells were stained in 96-well plates for flow cytometric analysis. Unspecific bindings were blocked by incubation with an unlabeled CD16/32-antibody (eBioscience) for 15 minutes on ice. Afterward, surface markers were stained with the respective primary and secondary antibodies for 15-20 minutes on ice and in the dark. The AnnexinV/PI-staining was performed using the “Annexin Apoptosis Detection Kit APC “(eBioscience) following the manufacturer’s protocol with minor alterations (25µl Staining solution with AnnexinV-APC 1:100 per sample; PI 1:200). Proliferation analysis was performed using the Proliferation Dye eFluor™ 670 (eBioscience) according to the manufacturer’s protocol. Stained cells were acquired using a Gallios flow cytometer (Beckman Coulter). Raw data were analyzed using Kaluza (Beckman Coulter, Krefeld, Germany) software. The following antibodies were used for flow cytometric stainings: From BD Pharmingen CD19 APC-Cy7 (Cat# 557655; Clone 1D3) and αCD5 PE (Cat# 553023; Clone 53-7.3); from Vector PNA FITC (Cat# FL-1071); from BioLegend CD138 Brilliant Violet 421 (Cat# 142508; Clone 281-2); from eBioscience CD11b FITC (Cat# 11-0112-85; Clone M1/70), CD95 PE (Cat# 12-0951-83; Clone 15A7), GL7 eFluor660 (Cat# 50-5902-82; Clone GL-7), TACI/CD267 PE (Cat# 12-5942-81; Clone eBio8F10-3), CD23 FITC (# 11-0232-82; Clone B3B4), CD21 biotinylated (Cat# 133-0211-82; Clone eBio8D9), BAFF-R APC (Cat# 17-5943; Clone ebio7h22-e16) and Apotracker™ Green (Cat#427401); from Jackson ImmunoResearch Streptavidin Cy5 (Cat# 016-170-084).

### Magnetic- or flow cytometric B cell isolation and *in vitro* culture

To isolate splenic follicular or marginal zone B cells by FACS, cell suspensions were stained as described for flow cytometric analysis in PBS- 2% FCS. Cells were stained in 15 mL reaction tubes. The solutions’ volumes were adjusted according to the used cell numbers. Cells were stained with CD19 Brilliant Violet 421 (BioLegend), CD23 PE (BioLegend), CD21 biotinylated (eBiosciene) and Streptavidin Cy5 (ImmunoResearch) and isolated with a purity >99% with the MoFlo cell sorter (Beckman Coulter). In addition, splenic naive B cells were isolated by magnetic cell sorting using the “EasySep™ Mouse B Cell Isolation Kit” from STEMCELL according to the manufacturer’s protocol. Isolated splenic B cells were cultured in complete RPMI1640 with 10% FCS and 10 µg/mL LPS with a density of 2x10^5^ cells/mL (37°C, 5% CO_2_).

### Fluorescence activated cell sorting for PCR analysis

To isolate splenic and peritoneal B cell populations by FACS, cell suspensions were stained as described for flow cytometric analysis in PBS- 2% FCS. Cells were stained in 15 mL reaction tubes. The solutions’ volumes were adjusted according to the used cell numbers. The following antibodies were used for flow cytometric stainings: From Biolegend CD19 BV510 (Cat#115545, Clone 6D5), CD138 PE-Cy7 (Cat# 142514, Clone 281-2), CD21/25 BV421 (Cat# 123421, Clone 7E9), CD23 APC.Cy7 (Cat# 101629, Clone B3B4), CD11b BV510 (Cat# 101263, CloneM1/70); from eBioscience CD19 AF647 (Cat# 51-0193-82, Clone eBio1D3), B220 PerCP.Cy5.5 (Cat# 45-0452-80, Clone Ra3-6b2), TACI APC (Cat# 17-5942, Clone eBio8F10-3), CD5 FITC (Cat# 53-7.3, Clone 53-7.3); from BD CD93 PE (Cat# 558039, Clone AA4.1).

### PCR

To analyze the DGCR8-alleles by PCR, genomic DNA was isolated from 5x10^4^ cells of the respective cell populations using the SampleIn™ Direct PCR Kit (highQu, Cat# DPK0101) and the following gene-specific primers in a PCR: 5’-GATATGTCTAGCACCAAAGAACTCC-3’ and 5’- GATCTCAGTAGAAAGTTTGGCTAAC-3’. For the loxP-flanked exon 3, a fragment with 730bp is expected, for the wildtype allele, a 500bp fragment, and the deleted allele, a 120bp fragment.

### RNA isolation

RNA, including microRNAs, were isolated using the “miRNeasy Micro Kit” (cell numbers ≥ 6 x 10^4^, Qiagen, Cat# 217024). FACS-isolated FO or MZ B cells were directly sorted into the Qiazol Lysis Reagent (700 µl final volume). The samples were stored at –70°C until further processing. Thawed samples were vortexed for 1 minute and incubated at RT for 5 minutes before further processing following the manufacturer’s manual. “On-column DNase digest” was performed according to the manufacturer’s manual. RNA concentrations and purity (absorption at 260 nm and a ratio of 260/280 of ~2.0, respectively) were determined using the NanoDrop ND-1000 (Peqlab).

### cDNA synthesis and TaqMan^©^ qRT-PCR

Isolated miRNAs were transcribed in PCR templates (cDNA) using the “TaqMan MicroRNA Reverse Transcription Kit” (Applied Biosystems, Cat# 4366597). 5 µl RNA (2ng/µg) and 3 µl of 5x Primer stock (ThermoFisher Scientific, miR-29a-3p: Cat# 002112; miR-16-1: Cat# 000391 or RNU6B Cat# 001973) were added to 7 µl master mix (0.15 μl dNTP, 1 μl transcriptase, 1.5 μl buffer, 0.2 μl RNase inhibitor and 4.15 μl RNAase-free water) for conversion of mature microRNAs to cDNAs. Mixtures were incubated in a PCR machine for 30 minutes at 16°C, 30 minutes at 42°C and 5 minutes at 85°C. The cDNA preparation was then pre-diluted 1:5 with RNAase-free water and quantified using the “TaqMan^©^ qPCR analysis TaqMan Universal Master Mix II” (Invitrogen, Cat# 4427788). For each reaction, 5 µl cDNA (1:5), 0.75 µl miRNA-specific probe mix (20x), 7.5 µl master mix and 1.75 µl RNAse-free water were mixed in 96-well plates (Thermo Scientific, Cat# AB-1100) and covered with “adhesive qPCR Plate Seals” (Thermo Scientific, Cat# AB-1170). Isolated mRNA to detect Bim was transcribed in PCR templates (cDNA) using the “RevertAid First Strand cDNA synthesis Kit” (Thermo Fischer Scientific, Cat# K1621) and quantified using TaqMan^©^ Genexpression Assay (ThermoFischer Scientific, Bcl2l11: Assay ID Mm000437796_m1; β-Actin Assay ID Mm02619580_g1). For each reaction, 2 µl cDNA (2ng), 10 µl master mix (2x), 1 µl probe mix (20x) and 7 µl RNase free water were mixed in 96-well plates. TaqMan^©^ qRT-PCR analysis was performed in the “QuantStudio 1 Real-Time PCR System” (Applied Biosystems). Each sample was measured in triplicates. Reactions without the cDNA template (NTC) served as a negative control to validate the specificity of the reaction. The mean of the Ct-values (cycle threshold) was calculated for the triplicates of each sample. The mean-Ct of the housekeeping gene RNU6B was subtracted from the mean-Ct of the respective microRNA, while β-Actin mRNA served as a housekeeper gene for mRNA quantification (37). This value was used to calculate the ΔCt-values.

### ELISpot and ELISA

To identify the frequencies of antibody-secreting cells in the single-cell suspensions from the spleen or the bone marrow of mice, ELISpot analysis was performed in 96-well flat-bottom plates as described in (38). To analyze total Ig-secreting cells, the plates were coated with goat-α-mouse IgM, IgG or IgA (Southern Biotech), while TNP-BSA (load 5; LGC BioSearch Technologies) was used for the identification of TNP-specific antibody-secreting cells. Cell suspensions were incubated overnight. Alkaline phosphatase (AP)-coupled goat α-mouse IgG, IgM or IgA antibodies (Southern Biotech) were used as detection antibodies. 5-Bromo-4-chloro-3-indolyl phosphate p-toluidine salt (BCIP; SigmaAldrich) was used in the ESA substrate buffer for detection (ESA substrate buffer 10x: 100 mL 1,5M AMP pH 10.3; 0.75 mL 1M MgCl_2_; 0.152 mL Triton X-405; 1.5 mL NaN_3_ 10%; 47.6 mL H_2_O; adjust to pH 10.25 with HCl; filter and store at 4°C protected from light. ESA substrate solution: 50mL 10x ESA-substrate buffer; 500mg BCIP; 450mL H_2_O; stir for 1 h at room temperature protected from light; filter and store at 4°C protected from light for max. ~3 months). Spots representing single antibody-secreting cells were counted using the Immuno-SpotR^©^ Series 6 Ultra-V Analyzer from C.T.L. and analyzed with the C.T.L. Software BioSpotR^©^ ImmunoSpot 5.1.36. For detecting serum Ig by ELISA, 96-well plates were coated as described for ELISpot-analysis. As detection antibodies, either AP-coupled α-mouse-Ig (IgG, IgA or IgM) antibodies or HRP-coupled α-mouse-Ig (IgG, IgA or IgM) antibodies (Sothern Biotech) were used. For AP- and HRP-coupled detection antibodies, alkaline phosphatase yellow (pNPP) liquid substrate (Sigma) and TMB Substrate Reagent Set (BD Pharmingen) were used, respectively. ELISA plates were measured using SpectraMax 190 at 450 nm (HRP) or 405 nm (AP).

### Immune histology

For immune histological analysis, splenic tissue samples were frozen at -80°C in Tissue-Tek^©^ O.C.T. ^©^ (Sakura), and sections were generated at the Leica CM3050S cryostat. Then, spleen sections were fixed in acetone (-20°C) and stained with the respective primary and secondary antibodies or chemicals. For the analysis of GCs, PNA Rhodamine (Vector; #RL-1072), αIgD FITC (SouthernBiotech; #1120-92) and αKi67 APC (BioLegend; #652406; Clone 16A8) were used before the sealing with VectaShield (Vector).

### Quantification of DNA abundance on agarose gel

ImageJ was used to quantify the DNA abundance of a PCR-fragment on an agarose gel (39). To calculate relative abundance, values were normalized to the signal intensity of an adequate background section.

### Statistical analysis

Significances and p-values were determined using the GraphPad Prism software (GraphPad Software, La Jolla, CA, USA). Statistical tests were performed as indicated below each figure.

## Data availability statement

The raw data supporting the conclusions of this article will be made available by the authors, without undue reservation.

## Ethics statement

All animal experiments were performed according to institutional and national guidelines, and were approved by the state of Bavaria (Amt für Veterinärwesen und gesundheitlichen Verbraucherschutz der Stadt Erlangen, Erlangen, Germany; Regierung von Unterfranken, Würzburg, Germany).

## Author contributions

PD, SO, JM, SS, JC-R, MH, ER and KP performed experiments. PD, KP and H-MJ designed experiments. PD, SO and KP analyzed and visualized the data. PD, KP and H-MJ interpreted the data. JM, SS, WS and DM provided scientific input for data interpretation. H-MJ conceptualized the project. H-MJ and KP supervised the project. PD, KP and H-MJ wrote the manuscript. All authors contributed to the article and approved the submitted version.
