# Supplementary material for: The microRNA processing subunit DGCR8 is required for a T cell-dependent germinal center response
Source: Front Immunol. 2022 Dec 16;13:991347. doi: 10.3389/fimmu.2022.991347 (PMC9800915; doi:10.3389/fimmu.2022.991347)
Supplement: Supplementary file 1 [file DataSheet_1.pdf]

SUPPLEMENTAL INFORMATION

Daum *et al*.

The microRNA processing subunit DGCR8 is required for a T cell-dependent germinal center response

(DOI: 10.3389/fimmu.2022.991347)

SUPPLEMENTAL FIGURES

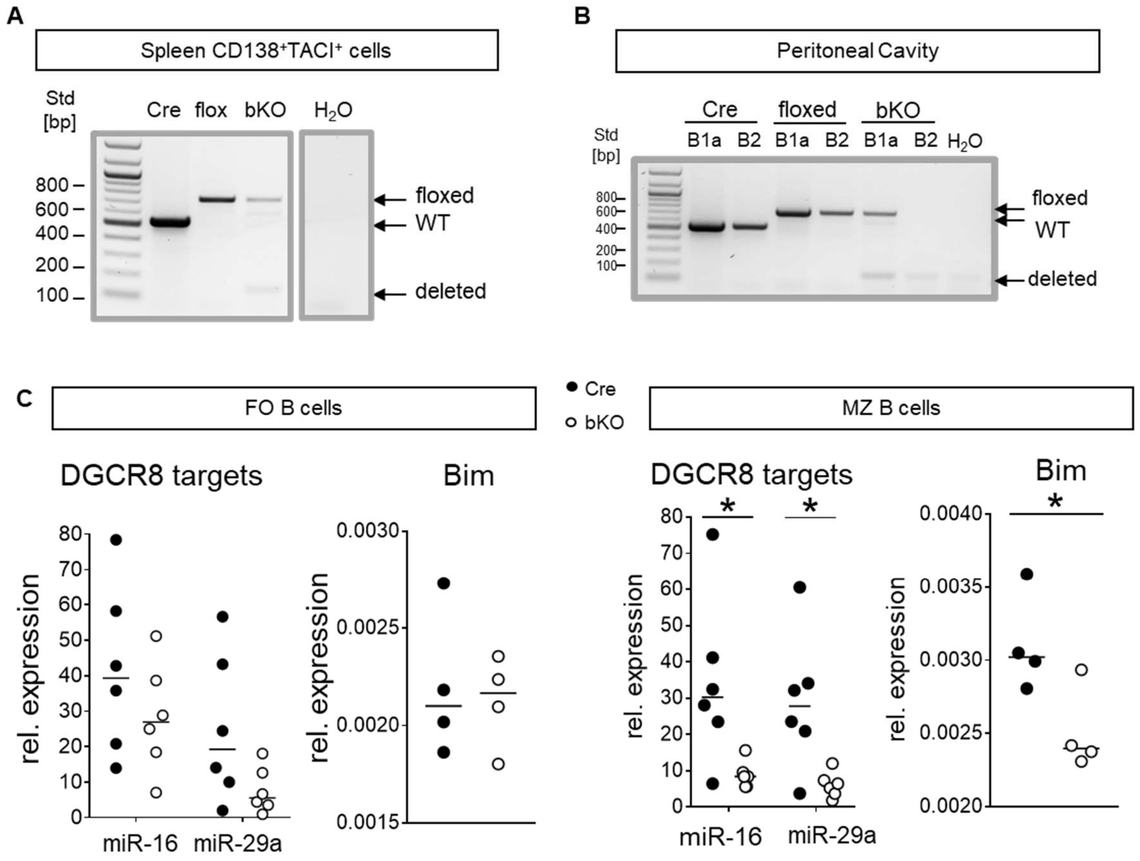

Supplemental Figure 1: Deletion efficiency in B cell populations of DGCR8-bKO mice. The DGCR8 locus was analyzed by PCR of flow cytometry-sorted **(A)** splenic ASCs ($CD138^+TACI^+$) and **(B)** B1a and B2 cells from the peritoneal cavity of DGCR8-bKO, Cre-only and flox-only (floxed) control mice, respectively. PCR products are ~530 bp for the wildtype, ~800 bp for the floxed and ~120 bp long for the Cre-deleted DGCR8 allele. **(C)** Splenic follicular (FO: $B220^+CD19^+AA4.1^-CD23^+CD21^+$) and marginal zone (MZ: $B220^+CD19^+AA4.1^-CD23^-CD21^+$) B cells of DGCR8-bKO mice and Cre control animals were isolated by FACS. microRNAs and mRNAs were isolated, reverse transcribed to cDNA and analyzed by TaqMan© qPCR. CT values of the housekeeping genes RNU6B (miRNA) or β-Actin (mRNA) were used to calculate ΔCT values. N=2, n=4-6. Each dot represents one mouse. Mann-Whitney test was used for statistical analysis. * $p<0.05$.

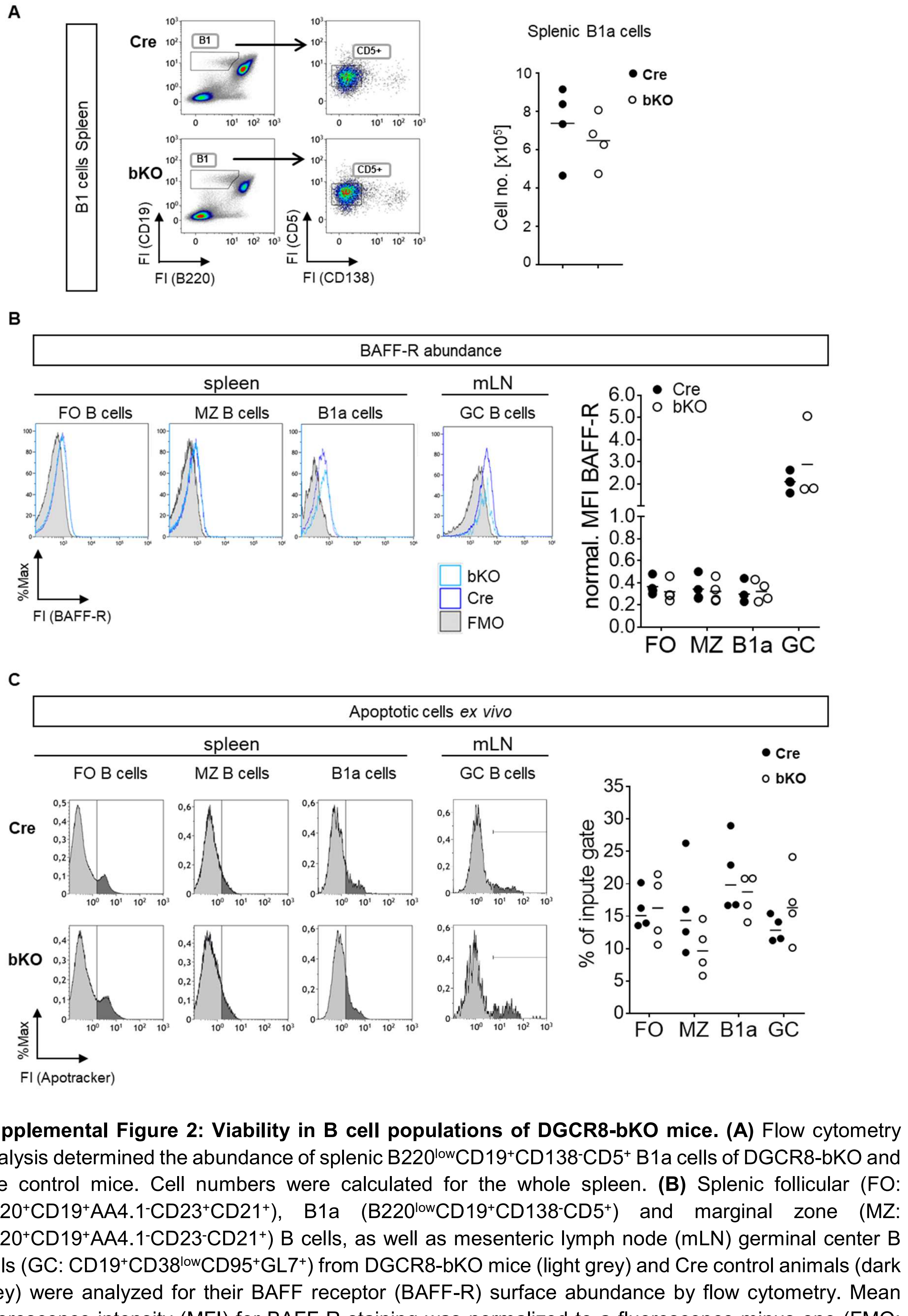

Supplemental Figure 2: Viability in B cell populations of DGCR8-bKO mice. (A) Flow cytometry analysis determined the abundance of splenic $B220^{low}CD19^{+}CD138^{-}CD5^{+}$ B1a cells of DGCR8-bKO and Cre control mice. Cell numbers were calculated for the whole spleen. **(B)** Splenic follicular (FO: $B220^{+}CD19^{+}AA4.1^{-}CD23^{+}CD21^{+}$), B1a ($B220^{low}CD19^{+}CD138^{-}CD5^{+}$) and marginal zone (MZ: $B220^{+}CD19^{+}AA4.1^{-}CD23^{-}CD21^{+}$) B cells, as well as mesenteric lymph node (mLN) germinal center B cells (GC: $CD19^{+}CD38^{low}CD95^{+}GL7^{+}$) from DGCR8-bKO mice (light grey) and Cre control animals (dark grey) were analyzed for their BAFF receptor (BAFF-R) surface abundance by flow cytometry. Mean fluorescence intensity (MFI) for BAFF-R staining was normalized to a fluorescence-minus-one (FMO; grey shade) control. **(C)** Splenic and mesenteric lymph node B cell populations defined by flow cytometry as described under (B) were analyzed for apoptosis *ex vivo* using an Apotracker dye. Bars indicate the median of n=4 biological replicates. Each dot represents one mouse. Mann-Whitney test was used for statistical analysis.
